# Collagenous gastritis: A cause of anemia and gastric nodularity in the pediatric population

**DOI:** 10.1002/jpr3.70156

**Published:** 2026-03-02

**Authors:** Megan Emiliani, Dyer Heintz, Marisa Izaguirre

**Affiliations:** ^1^ Department of Pediatrics University of Texas Dell Medical School Austin Texas USA; ^2^ Division of Pediatric Gastroenterology Dell Children's Medical Center Austin Texas USA

**Keywords:** iron deficiency, subepithelial collagen, vitamin B12

## Abstract

We present a case series of seven pediatric patients with collagenous gastritis (CG) with the aim of contributing data to existing literature about this rare disease to assist in the understanding and management of these patients. Gross nodularity seen on esophagogastroduodenoscopy and increased subepithelial collagen on biopsy were noted in the gastric body of all seven patients. Iron deficiency anemia was also diagnosed in all patients and did improve with iron supplementation. The most common symptoms were abdominal pain, fatigue, dizziness, and vomiting. Some patients seemed to have improvements in abdominal pain after use of antisecretory agents, such as proton pump inhibitors and histamine (H2) antagonists. All of the patients in our study had complete resolution of symptoms, but none showed improvements histologically. Even three patients who were trialed on oral or topical budesonide exhibited neither gross nor histologic improvements. Further clinical trials are needed to identify effective therapeutic strategies for CG that can result in long‐term histological improvement.

## Abstract (highlights)

**What is Known:**

- Pediatric‐onset collagenous gastritis (CG) classically presents with abdominal pain and iron deficiency anemia (IDA).
- There is no definitive cure for CG, nor are there standard treatment options. Various interventions seem to have limited efficacy.
- Patients may experience resolution of symptoms despite no improvements demonstrated histologically.
- Long‐term iron supplementation appears to control the associated IDA.

**What is New:**

- The disease may extend into the duodenal bulb, with duodenal biopsy showing inflammation and increased sub epithelial collagen.
- One patient presented with a normocytic anemia and B12 deficiency rather than iron deficiency.
- Treatment with budesonide did not improve endoscopic or histologic findings.

## 1 INTRODUCTION

Collagenous gastritis (CG) may not be the first diagnosis that comes to mind when a child presents with fatigue, severe iron deficiency anemia, and gastrointestinal symptoms, but it should be considered in the differential. CG is a rare condition characterized by subepithelial deposits of collagen bands and inflammation in the gastric mucosa. The pathogenesis of CG remains poorly understood, however some studies propose the etiology may be immune or autoimmune.^1^, ^2^ The two phenotypes of CG that have been identified include pediatric‐onset and adult‐onset. Pediatric‐onset CG does not typically involve the colon, whereas adult‐onset differs in that it involves subepithelial collagen bands in the stomach, duodenum, and colon.^3^ Previous studies have shown that the most common symptoms in pediatric‐onset CG include abdominal pain, vomiting, and anemia.^4^ However, adult‐onset CG tends to present with chronic diarrhea and weight loss. Currently, no definitive treatment or management guidelines exist for children with CG. The medications and interventions commonly used have limited benefit in treating these patients and are usually supportive.^5^ Nonetheless, recent evidence suggests topical steroids can result in clinical and histologic improvement in these patients, though this study included mostly adults.^3^ In this case series, we aim to report the demographic and clinical features as well as diagnostic findings and treatment outcomes in a series of cases of pediatric‐onset CG, with the ultimate goal of working towards an effective standardized approach for management.

## 2 METHODS

Through retrospective chart review of the electronic medical record, we identified all patients under 18 years old with the diagnosis of “CG” between the years 2019–2025 at Dell Children's Medical Center in Austin, TX. The diagnosis was confirmed by the presence of a prominent subepithelial collagen greater than 10 µm in thickness on gastric biopsy. We manually collected demographic data including age at diagnosis, sex, and race/ethnicity. Clinical features and findings including initial presentation, hemoglobin level, endoscopy, and pathology results were also noted. Lastly, we evaluated courses of treatment with comparisons of outcomes after treatment.

### 2.1 Ethics statement

This retrospective study met criteria for exemption from IRB review by the University of Texas at Austin Institutional Review Board, and it received site approval from Ascension Seton.

## 3 RESULTS

A total of seven patients were identified and met inclusion criteria. Clinical characteristics for each patient are depicted in Table 1. The median age at diagnosis was 12 ± 4.9 years (3–14 years). The most common presenting symptoms included abdominal pain (4/7 or 57%), followed by fatigue (3/7 or 43%), dizziness/syncope (3/7 or 43%), and vomiting (3/7 or 43%). All patients had moderate to severe anemia, with hemoglobin levels ranging between 2.2 and 11.1 g/dL, and all except one were iron deficient. Interestingly, the one patient who was not iron deficient actually had a vitamin B12 deficiency. None of the patients presented with hematochezia or melena, but four out of the seven (57%) tested positive for fecal occult blood.

**Table 1:** Patient demographics, symptoms, and findings at initial presentation.

Age (years)	Sex	Ethnicity, Race	Symptoms	Hgb level (g/dL)	Endoscopy findings	Pathology findings
13	F	Hispanic, White	Abdominal pain, dizziness	5.0	Nodularity and friability in gastric cardia, fundus, body	Subepithelial collagenous bands in gastric body; Chronic gastritis in body and antrum; Focal gastric foveolar metaplasia in duodenal bulb
3	M	Non‐hispanic, White	Dizziness, fatigue, pallor, weight loss, vomiting	2.2	Nodularity, friability, and scant old blood in gastric fundus, cardia, and body; Nodularity in duodenal bulb	Prominent subepithelial collagen and chronic gastritis in stomach; Normal duodenum
14	M	Non‐hispanic, White	Abdominal pain	11.1	Moderate nodularity, erythema, and friability in gastric body and fundus	Increased subepithelial collagen and chronic gastritis in body and antrum; Normal duodenum
12	F	Non‐hispanic, White	Abdominal pain, syncope, pallor, constipation, dark stools	8.6	Moderate erythema and diffuse plaque‐like appearance in entire stomach with one small erosion	Prominent subepithelial collagen in stomach and increased lamina propria eosinophils; Normal duodenum
13	M	Asian	Fatigue, vomiting	4.9	Moderate edema, erythema, friability, and scant dried blood in gastric body	Increased subepithelial collagen and mild chronic gastritis in body and antrum; Increased subepithelial collagen in duodenal bulb
12	M	Non‐hispanic, White	Fatigue, pica	9.0	Nodularity and pseudopolyps as well as severe erythema and friability localized in gastric fundus and body	Increased subepithelial collagen in gastric body with surface erosion; Normal duodenum
9	F	Hispanic, White	Abdominal pain, nausea, vomiting	6.7	Moderate erythema, erosion, friability, and granularity in gastric body; Mild erythema and erosion in fundus and antrum; Nodularity in duodenal bulb	Prominent subepithelial collagen in stomach and Increased lamina propria eosinophils; Normal duodenum

Abbreviations: F, female; Hgb, hemoglobin; M, male.

On esophagogastroduodenoscopy (EGD), all patients exhibited nodularity visible in the stomach, which was either localized or diffuse. Two patients also had nodularity in the duodenal bulb. All of the patients had biopsies that revealed prominent subepithelial collagen in the stomach. Two patients also had histologic abnormalities in the duodenal bulb on their initial biopsies even though they appeared normal on EGD. One of those patients had a subtle increase in subepithelial collagen, while the other had focal gastric metaplasia in the duodenal bulb. Later in the disease course on repeat biopsy, a different patient developed subepithelial collagen deposition in the duodenal bulb that was not seen on initial biopsy. Interestingly, the two patients with visible nodularity in the duodenal bulb on EGD showed a normal duodenum histologically. All patients had colonoscopies given the severity of their anemia associated with either occult bleeding or chronic abdominal pain. All colonoscopies were unremarkable with normal colonic biopsies.

Four patients required packed red blood cell (PRBC) transfusions for severe anemia, and all patients were treated with oral iron supplementation. Two patients with recurrent severe anemia received intravenous iron. Again, one patient had normocytic anemia with B12 deficiency and normal iron studies. This patient was treated with both iron and B12 supplementation. Hemoglobin levels in all patients improved overtime after long‐term iron supplementation.

**Figure 1:**
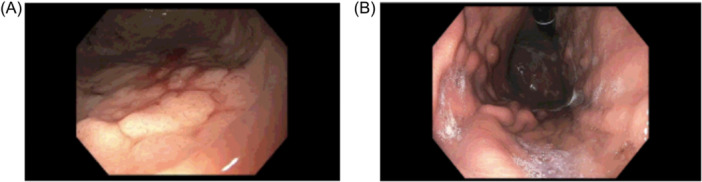
Endoscopic images of the stomach. Panel (A) shows nodularity and inflammation with scant bleeding in the gastric body from initial endoscopy of one of the patients. Panel (B) shows endoscopic findings of the gastric body 40 months after the initial scope. This patient had completed a 3‐month course of treatment with oral budesonide and long‐term oral iron supplementation. The follow up imaging (B) exhibits continued nodularity and inflammation despite treatment.

Five patients were treated with antisecretory medications, four with proton pump inhibitors and one with histamine (H2) antagonists. It is unclear if these were directly associated with symptom resolution in patients. Four patients were treated with a 6–8 week course of oral budesonide, although none were found to have endoscopic or histological improvement. Endoscopic findings of one patient before and after treatment are shown in Figure 1. One patient trialed a gluten‐free diet, but it did not seem to improve symptoms nor lead to changes on biopsy. The time frame in which endoscopies were repeated varied significantly. Three patients had a repeat EGD with biopsies 4–6 months after initial diagnosis, and all were unchanged despite two of those patients completing a course of budesonide. Three patients had a repeat EGD with biopsies 1–3 years after initial diagnosis, and none were improved from prior. In one of those patients, disease actually progressed into the duodenal bulb where it was not seen previously, with nodularity and erythema seen endoscopically and duodenal biopsies showing inflammation with increased subepithelial collagen. All of the patients except for two in our study had repeat upper endoscopies at some point in time.

## 4 DISCUSSION

Pediatric patients who have overt nodularity in the gastric mucosa on EGD should be biopsied to assess for CG since this seems to be the most common sign for the diagnosis, as also reported in other studies.^6^, ^7^ Biopsy revealing subepithelial collagen deposition greater than 10 μm in thickness confirms the diagnosis.^7^ All of our patients had nodularity and prominent subepithelial collagen in the gastric body, with variable sparing in other portions of the stomach. A large review of the literature noted that while collagen band distribution varied, most cases demonstrated collagen thickening diffusely throughout the stomach or solely in the gastric body.^7^ None of the patients had collagenous colitis, which is already known to be uncommon in children compared to adults.^8^, ^9^ However, three patients in our study did develop duodenitis in the duodenal bulb two on initial biopsy and one on repeat biopsy 1 year after diagnosis. Only two of the biopsies in our case series demonstrated an increase in subepithelial collagen. Most cases of pediatric CG report collagen deposition limited to the stomach.^3^, ^8^ There are limited case reports of children with collagenous gastroduodenitis.^10^, ^11^, ^12^, ^13^, ^14^, ^15^ Patients were diagnosed with collagenous duodenitis based on the presence of a subepithelial collagen band >10μm thick in the duodenum. Three of these cases revealed the endoscopic appearance of cobblestoning or nodularity in the duodenal bulb.^10^, ^11^, ^12^, ^13^, ^14^, ^15^ All cases had collagen deposition in the duodenal bulb with normal second and third portions of the duodenum. Similarly to one of our patients, one of the cases had a normal duodenal biopsy initially and went on to develop collagenous duodenitis on repeat biopsy.^15^ Although pediatric‐onset disease is not always limited to the stomach, it almost always involves the stomach. The differences in locations of disease involvement, especially in adult versus pediatric‐onset, may suggest differences in the etiologies of both types. Conversely, some have proposed the pediatric and adult phenotypes may be part of a continuous spectrum.^9^ A recent gene expression profiling study found enhanced expression of T helper 1 cell (Th1) and T helper 2 cell (Th2) cytokines in a cohort with isolated gastric CG, which could support a role for environmental or allergic factors.^16^ Still, the pathogenesis of this condition remains poorly understood.

Anemia appears to be due to occult gastrointestinal bleeding as evidenced by over half our patients with positive fecal occult blood tests. All but one patient had iron deficiency, which may be due to the disease state being caught early in the course when iron stores were not depleted yet. That patient was found to be vitamin B12 deficient. There are no other case reports of patients with CG having an associated B12 deficiency, so it is unclear if this is directly related to CG. It may be in a similar mechanism to autoimmune atrophic gastritis, in which there is malabsorption of vitamin B12 due to the atrophy of gastric parietal cells producing intrinsic factor.^17^ One study suggests CG can, uncommonly, be associated with an atrophic gastritis inflammatory pattern.^18^ However, there was no evidence of atrophy on our patient's biopsies. The patient did follow a primarily pescatarian diet, which may have contributed. Hematology was consulted on this patient, and they suggested the normocytic anemia was caused by a combination of GI blood loss along with macrocytosis from the B12 deficiency. The patient's B12 levels normalized after 2 months of B12 supplementation. All patients benefited from long‐term iron supplementation, which treated the anemia and kept hemoglobin levels stable.

Antisecretory medications were used in most of our patients and may have aided in resolution of abdominal pain. Unlike one recent study which showed a histologic response to oral budesonide, none of the three patients who had biopsies after steroid treatment showed notable improvements.^3^ Another large case series did have one pediatric patient who achieved improvement of histologic features after treatment with oral prednisolone and long‐term azathioprine.^9^ Azathioprine is not historically used for pediatric patients with CG, so further randomized clinical trials are needed to determine its effectiveness. Further studies are needed to evaluate the long‐term complications of poorly controlled CG in the pediatric population. There is certainly a need to raise awareness to clinicians, pathologists, and researchers about this rare disease.

## 5 CONCLUSION

Our case series highlights interesting findings of pediatric‐onset collagenous gastritis including extension of disease into the duodenal bulb, potential association with B12 deficiency, and an absence of endoscopic or histologic improvements after treatment with budesonide. Having a larger compilation of data on this rare disease can help us better understand the pathophysiologic mechanism of pediatric‐onset CG in order to develop effective therapeutic strategies.

## CONFLICT OF INTEREST STATEMENT

The authors declare no conflicts of interest.

## References

[1] Käppi T , Wanders A , Wolving M , et al. Collagenous gastritis in children: incidence, disease course, and associations with autoimmunity and inflammatory markers. Clin Transl Gastroenterol. 2020;11(8):e00219. 10.14309/ctg.0000000000000219 32955189 PMC7431242

[2] Nielsen OH , Riis LB , Danese S , Bojesen RD , Soendergaard C . Proximal collagenous gastroenteritides: clinical management. A systematic review. Ann Med. 2014;46(5):311‐317. 10.3109/07853890.2014.899102 24716737

[3] Choung RS , Sharma A , Chedid VG , Absah I , Chen ZE , Murray JA . Collagenous gastritis: characteristics and response to topical budesonide. Clin Gastroenterol Hepatol. 2022;20(9):1977‐1985.e1. 10.1016/j.cgh.2021.11.033 34864160

[4] Beinvogl BC , Goldsmith JD , Verhave M . Pediatric collagenous gastritis: clinical and histologic outcomes in a large pediatric cohort. J Pediatr Gastroenterol Nutr. 2021;73(4):513‐519. 10.1097/MPG.0000000000003212 34173792

[5] Pinis M , Ziv‐Sokolovskaya N , Kori M . Collagenous and lymphocytic gastritis in pediatric patients. A single‐center experience observing an increase in diagnosis in recent years. Scand J Gastroenterol. 2024;59(10):1144‐1150. 10.1080/00365521.2024.2395858 39206869

[6] Lee YJ , Lee M , Kim D , Lee S , Hong J . Three case reports of collagenous gastritis in children: lessons for an endoscopic and histologic approach to mucosal nodularity of the stomach. Medicine. 2019;98(11):e14870. 10.1097/MD.0000000000014870 30882690 PMC6426568

[7] Li J , Tanager K , Setia N . Collagenous gastritis: clinical features, histologic correlates and unanswered questions. Histopathology. 2025;87:789‐801. 10.1111/his.15542 40911031 PMC12605687

[8] Kamimura K . Collagenous gastritis: review. World J Gastrointest Endosc. 2015;7(3):265‐273. 10.4253/wjge.v7.i3.265 25789098 PMC4360446

[9] Matta J , Alex G , Cameron DJS , Chow CW , Hardikar W , Heine RG . Pediatric collagenous gastritis and colitis: a case series and review of the literature. J Pediatr Gastroenterol Nutr. 2018;67(3):328‐334. 10.1097/MPG.0000000000001975 29601434

[10] Koide T , Mochizuki T , Kawai N , et al. Collagenous gastroduodenitis with recurrent gastric ulcer in 12‐year‐old girl. Pediatr Int. 2015;57(4):754‐757. 10.1111/ped.12615 26011716

[11] Agrawal P , Bhattar K , Rojas C , Larson J . Pediatric collagenous gastroduodenitis: a rare cause of iron‐deficiency anemia. Cureus. 2024;16(11):e72939. 10.7759/cureus.72939 39498423 PMC11532023

[12] Kang B , Um SH , Yun J , Kim HK , Choe BH , Lee YM . Collagenous gastroduodenocolitis in a Korean adolescent: first pediatric case report in Asia. Transl Pediatr. 2021;10(11):3096‐3103. 10.21037/tp-21-342 34976776 PMC8649595

[13] Billiémaz K , Robles‐Medranda C , Le Gall C , et al. A first report of collagenous gastritis, sprue, and colitis in a 9‐month‐old infant: 14 years of clinical, endoscopic, and histologic follow‐up. Endoscopy. 2009;41(suppl 2):E233‐E234. 10.1055/s-2008-1077440 19757370

[14] Leiby A , Khan S , Corao D . Clinical challenges and images in GI. Gastroenterology. 2008;135(1):17‐327. 10.1053/j.gastro.2008.06.007 18555018

[15] Beinvogl BC , Goldsmith JD , Arumugam R , et al. Pediatric collagenous gastroenterocolitis successfully treated with methotrexate. Case Rep Pediatr. 2020;2020:1929581. 10.1155/2020/1929581 32181040 PMC7060430

[16] Liu Q , Wang Y , Harpaz N . Coexisting Th1 and Th2 cytokines in patients with collagenous gastritis and implications for its pathogenesis. J Pediatr Gastroenterol Nutr. 2024;78(2):231‐240. 10.1002/jpn3.12109 38374564

[17] Castellana C , Eusebi LH , Dajti E , et al. Autoimmune atrophic gastritis: a clinical review. Cancers. 2024;16(7):1310. 10.3390/cancers16071310 38610988 PMC11010983

[18] Arnason T , Brown IS , Goldsmith JD , et al. Collagenous gastritis: a morphologic and immunohistochemical study of 40 patients. Mod Pathol. 2015;28(4):533‐544. 10.1038/modpathol.2014.119 25234289

